# Nevus Lipomatosus Superficialis with a Folliculosebaceous Component: Report of 2 Cases

**DOI:** 10.4061/2011/105973

**Published:** 2011-04-18

**Authors:** Eugenia Bancalari, Diego Martínez-Sánchez, Juan C. Tardío

**Affiliations:** ^1^Department of Pathology, Hospital Universitario de Fuenlabrada, Camino del Molino 2, 28942 Fuenlabrada, Madrid, Spain; ^2^Department of Dermatology, Hospital Universitario de Fuenlabrada, Camino del Molino 2, 28942 Fuenlabrada, Madrid, Spain

## Abstract

Nevus lipomatosus superficialis (NLS) is an uncommon hamartomatous lesion of the skin characterized by the presence of clusters of mature fat cells among the collagen bundles of the dermis. Usually, the number of adnexal structures is reduced in NLS as compared to the normal adjacent skin, but their morphology is not altered. Nevertheless, in some instances, associated pilar abnormalities have been reported. We here report 2 cases of NLS with disorganized pilosebaceous units. The follicular structures were dilated and showed infundibular type keratinization and there were numerous mature sebaceous lobules radiating from them resembling the histology of a folliculosebaceous cystic hamartoma (FSCH) or a sebaceous trichofolliculoma. Only 2 cases of NLS with FSCH have been previously reported. Our cases represent examples of a very rare hamartomatous cutaneous lesion composed of mesenchymal and epithelial components.

## 1. Introduction

Nevus lipomatosus superficialis (NLS) is an uncommon cutaneous hamartoma consisting of flesh-colored or yellowish soft nodules or papules with smooth or wrinkled surface, which are histologically composed of groups of mature fat cells located among the bundles of dermal collagen. Two clinical forms are recognized: the classical and the solitary one. In the first, there are multiple papules and nodules grouped in a zonal pattern forming a plaque or a cerebriform mass. They present at birth or in the first two decades of life and are usually located on the buttocks, lower back, or upper thighs. On the contrary, the solitary type can develop in adults and shows a wider distribution in the skin [1]. NLS has neither a predominant sex incidence nor a familial tendency. The patients generally carry no other congenital defects. 

Usually, the number of adnexal structures is reduced in NLS as compared to the normal adjacent skin but their morphology is not altered [1]. However, some cases of NLS with pilar anomalies have been reported, such as abortive hair germ-like structures [1], hypertrophic pilosebaceous units [2], perifollicular fibrosis [3], fibrofolliculomas [4], and folliculosebaceous cystic hamartomas (FSCHs) [5, 6].

We herein describe 2 cases of NLS with abnormal folliculosebaceous structures, a very rare hamartomatous lesion of the skin with mesenchymal and epithelial components.

## 2. Case Report

**Case 1**

**Figure 1:**
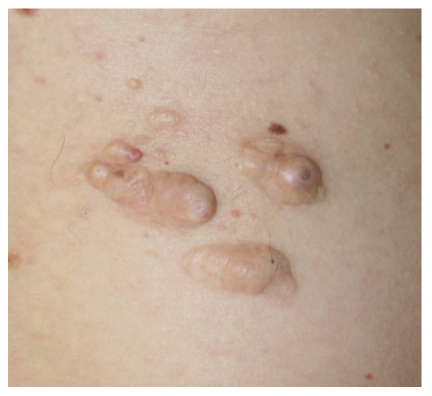
Case 1. A skin-colored cutaneous plaque composed of confluent nodules on the right lumbar region.

**Figure 2:**
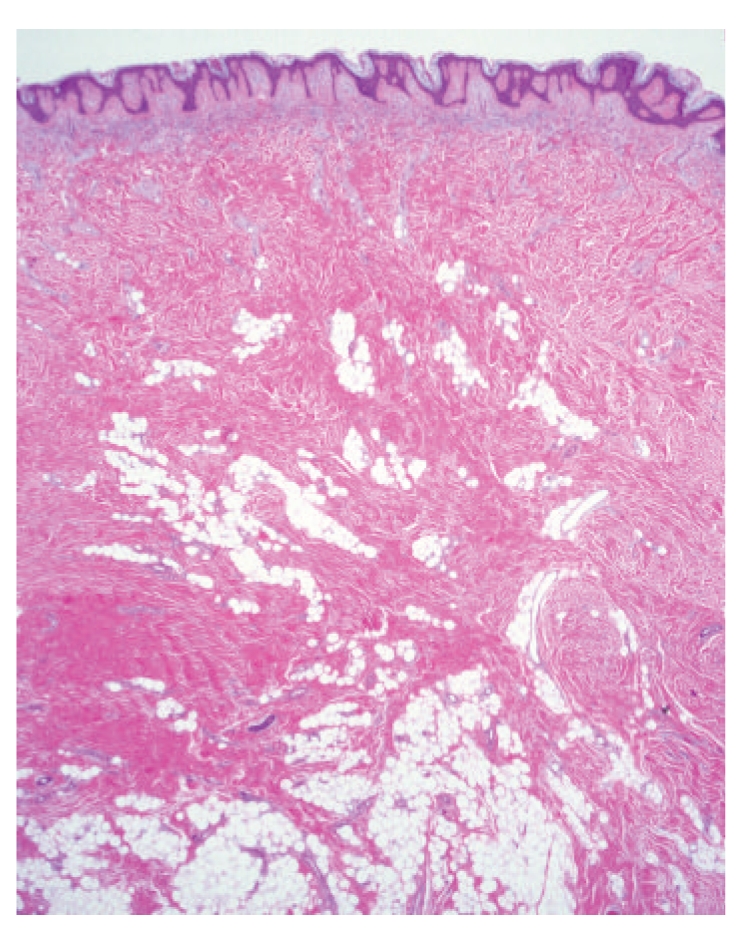
Case 1. A nodule formed by clusters of mature fat cells amongst thick collagen dermal bundles. This nodule lacked an epithelial component.

**Figure 3:**
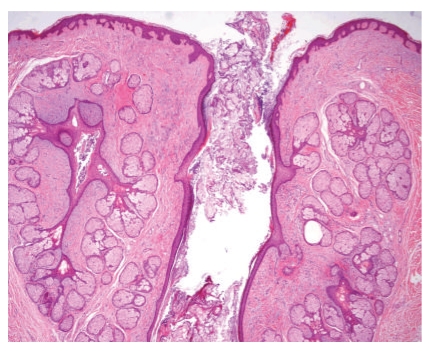
Case 1. Two follicular structures with numerous radiating mature sebaceous glands. One of the follicles is dilated and connects with the epidermal surface.

**Figure 4:**
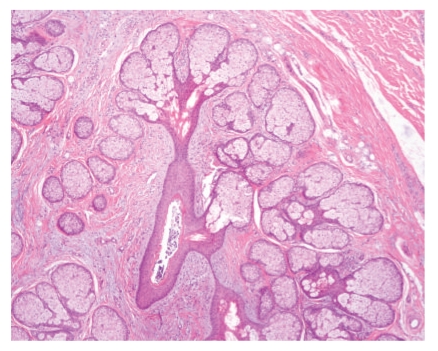
Case 1. A folliculosebaceous unit embedded in a mucinous stroma with small vessels and scarce mature fat cells. The lesion is separated from normal dermis by a linear cleft (*on the right*).

A 33-year-old male presented with a 90 × 40 mm, asymptomatic, slowly growing cutaneous plaque on his right lumbar region, which arose in childhood. It was composed of multiple, confluent, skin-colored nodules (Figure 1). Some of them were centered by comedo-like plugs. He had no other skin or internal abnormalities. The clinical diagnosis was connective tissue hamartoma with a cystic component versus lymphangioma. An 11 × 7 mm skin biopsy was performed. Histology showed a keratogranulomatous foreign body giant cell inflammatory infiltrate within a fibrous dermis. Due to the lack of a definitive diagnosis, a partial surgical resection, consisting of a 55 × 32 mm cutaneous fragment containing several nodules was performed. The cut surface of the nodules was yellowish white, and there were dilated follicular structures in some of them. Histologically, the nodules were composed of fibrous tissue, with irregularly arranged collagen bundles and sparse elastic fibers, and clusters of mature fat cells (Figure 2). Most of them also contained disorganized pilosebaceous units embedded in a paucicellular fibrous stroma. The follicular structures showed an infundibular-type keratinization, being occupied by keratin scales and frequently cystically dilated. They were located at different levels of the dermis and in the superficial subcutaneous tissue, and some were connected with the epidermal surface. Numerous mature sebaceous glands radiated from the follicular structures (Figure 3). The stroma surrounding the pilosebaceous units contained scarce fibroblasts, mucin deposits, small blood vessels, and occasional mature fat cells. Some of the cysts were ruptured, causing a foreign body inflammatory response similar to that seen in the patient's previous biopsy. Linear clefts separated the folliculosebaceous units and their stroma from the surrounding dermis (Figure 4). A minority of the nodules lacked epithelial structures. The epidermis showed slightly elongated rete ridges and a band of mucin could be observed under the epidermis throughout the entire lesion. This case was diagnosed as a classical NLS with FSCH.

**Case 2**

**Figure 5:**
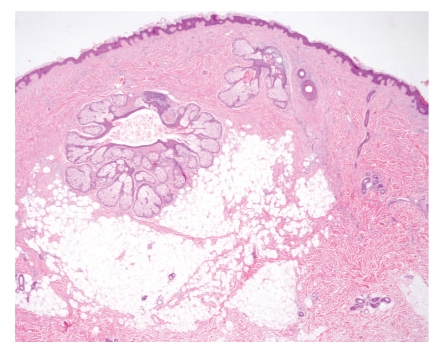
Case 2. Low magnification view of the lesion composed of confluent groups of mature fat cells among the dermal collagen and a distorted pilosebaceous structure.

**Figure 6:**
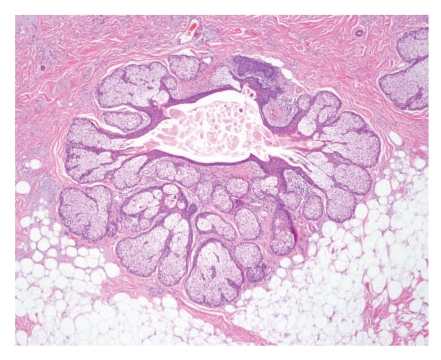
Case 2. Numerous sebaceous lobules and a small hair follicle *(on the top)* arising from a cystic cavity limited by squamous epithelium with epidermoid keratinization.

A 51-year-old male visited our Dermatology Department for a 6 mm nodular dermal lesion on his right forearm. It had been present for several years. The clinical diagnosis was leiomyoma. The lesion was entirely excised. On cutting, it was observed that it occupied the whole dermis and showed a yellowish white color, a bland consistency, and a poorly demarcated margin. Histologically, it consisted of numerous, confluent groups of mature fat cells located among the dermal collagen bundles (Figure 5). Small perivascular and interstitial deposits of mucin could be observed throughout the lesion. An abnormal folliculosebaceous structure was found in the reticular dermis. It was centered by a dilated lumen, occupied by keratin, sebaceous material, and some demodex. Mature sebaceous lobules and small follicles arose from the central cavity (Figure 6). This pilosebaceous unit opened to the epidermal surface. The epidermis did not exhibit anomalies. The diagnosis was solitary NLS with sebaceous trichofolliculoma (TF). The patient presented with two other cutaneous lesions of polypoid morphology, which measured 18 and 6 mm and were located on the right buttock and left lower back, respectively. Both were also surgically resected. The diagnoses were NLS, solitary type, in the former and skin tag in the latter.

## 3. Discussion

We herein report 2 cases of cutaneous hamartoma consisting of clusters of mature fat cells located among the collagen bundles of the dermis and disorganized infundibular structures with numerous radiating well-developed sebaceous glands. We consider both cases as examples of NLS with a folliculosebaceous component. A follicular component in an NLS is a very unusual event, which can clinically be suggested by the presence of umbilicated papules in the lesion [4]. 

Our case 1 resembled a giant FSCH. FSCH is a rare developmental cutaneous abnormality formed by dilated and distorted folliculosebaceous units embedded in a stroma containing spindle and stellate fibroblasts, and variable amounts of mature fat cells, blood vessels, nerves, and mucin. FSCH typically presents as a solitary small papulonodular lesion in the central face or scalp of adults [7]. Other locations and giant forms have occasionally been reported. Unlike the classical form, the giant variant of FSCH is usually located in the genital area or in other extrafacial regions and can present at birth or during childhood [8–10]. Some nodules of our case 1 lacked epithelial structures and were entirely composed of clusters of mature fat cells located amongst irregularly arranged bundles of dermal collagen, corresponding to the morphology of an NLS. Due to the histology of these nodules, we interpret this lesion as an NLS with FSCH and not as a giant FSCH.

Our case 2 must be differentiated from a skin tag with fat cells and from a sebaceous TF. The presence of a distorted folliculosebaceous structure allows the differential diagnosis with the former. Sebaceous TF is a rare cutaneous hamartoma that presents as a centrally depressed small lesion on the nose and, rarely, on other areas of the head, neck, and genitals. Histologically, it consists of a cystic central cavity connected to the epidermis and lined by squamous epithelium with infundibular keratinization. Sebaceous lobules and hair follicles in various stages of their growth cycle arise from the wall of the cyst [11]. Some authors think that sebaceous TF and FSCH are two different names for the same lesion, merely representing a late stage of TF [12]. Location on the forearm is extremely unusual for a sebaceous TF, our patient had another solitary NLS with a typical histology including fat cells in the papillary dermis [1], and most of the nodule of our case 2 consists of clusters of mature fat cells among the dermal collagen with focal presence of only one disorganized folliculosebaceous unit. We consider that these features favor our interpretation of this lesion as an NLS with sebaceous TF.

Two cases of NLS with FSCH have previously been published. The first one was reported by Brasanac and Boricic in 2005 and described as a 23 cm plaque on the sacral region of a 47-year-old female that had been present since birth. This lesion also contained dermoid cysts [5]. The second case, reported by Kang et al. in 2007, was that of a 36-year-old male with a 10 cm cerebriform mass on the sacral area of 25 years duration [6].

Since mesenchymal dermal components other than fat cells, such as collagen bundles, elastic fibers, fibroblasts, and blood vessels used to be altered in NLS, some authors consider this lesion as a type of connective tissue nevus [13, 14]. Cases of connective tissue hamartomas with altered epithelial elements have rarely been reported [15, 16]. Recently, a NLS with a 2p24 deletion has been published [17]. No other articles addressing cytogenetic aberrations in NLS can be found in the literature up-to-date. More studies are needed to reach conclusions about the role of the genetic abnormalities in the development of NLS and to help clarify the possible relationship between NLS and connective tissue nevus.

In conclusion, we here report 2 cases of NLS with disorganized folliculosebaceous structures, a very rare hamartomatous cutaneous lesion composed of mesenchymal and epithelial components.
